# Sociodemographic characteristics of healthy volunteers along with their experience, attitude and concerns of clinical trials in Wuhan, China

**DOI:** 10.1038/s41598-023-46979-z

**Published:** 2023-11-09

**Authors:** Hengyi Yu, Yinian Fang, Xinxin Qi, Kaifu Wang, Yongfang Lei, Donglin Zhang, Qian Chen, Dong Liu, Xiuhua Ren

**Affiliations:** 1 Department of PharmacyTongji Hospital, Tongji Medical College, Huazhong University of Science and Technology, Wuhan, 430030 China; 2 Phase I Clinical Trial Unit, Tongji Hospital, Tongji Medical College, Huazhong University of Science and Technology, Wuhan, 430030 China

**Keywords:** Medical ethics, Drug development

## Abstract

China has became the world’s second largest pharmaceutical market, and the number of her registered clinical trials exceeded 3000 in 2021. Although thousands of healthy volunteers are participating in a large number of clinical trials in this country, there is no report about the characteristics, recognition, attitude of Chinese healthy volunteers and their concerns of clinical trials. A questionnaire survey was designed and given to 324 healthy volunteers participating in clinical trials in Wuhan, China. Four important findings emerged from our data. First, young, single and less educated men constituted the majority of Chinese healthy volunteers. Second, differences between the male and female healthy volunteers were observed. Female healthy volunteers are supposed to face more challenges and pressure in life, be more cautious about the clinical trials and more concerned about their health and feelings than the male. Third, no sociodemographic characteristic was associated with poorly understanding of the protocol research content, which was subjectively evaluated. Fourth, more support from society/family and more positive media reports about the participation of healthy volunteers in clinical trials are badly needed. These findings would help us to get a better understanding of Chinese healthy volunteers as a group for protecting them and promoting drug development.

## Introduction

With its fast-growing economy and large patient populations, China has become a huge market for drugs over the past decade and surpassed Japan to be the world’s second largest pharmaceutical market by sales since 2018^1,2^. At the same time, China also wants to be an important innovator of pharmaceutical products. China was thought to be a good place to conduct clinical trials owing to its large patient populations, skilled clinical trial and laboratory professionals and project leaders, and a large network of hospitals qualified to conduct high standard clinical trials^3^. In recent years, with the strong support of national policy and improved drug regulatory reform after the publication of ‘State Council Circular No. 44’ on August of 2015^4^, China is striving to be at the forefront of clinical research and drug development^5^, and the number of clinical trials conducted in this country is increasing significantly^6^.

In 2021, the total number of registered clinical trials in China exceeded three thousand for the first time^7^, an increase of 29.1% compared with the registered clinical trials in 2020. Among the 3358 registered clinical trials, 2033 new drug clinical trials accounted for 60.5% and 1325 bioequivalence (BE) tests accounted for 39.5%^7^. Among the 2033 new drug clinical trials, 872 phase I clinical trials accounted for the highest proportion of 42.9%^7^.

It is known that phase I clinical studies and BE tests of investigational drugs are mainly conducted in healthy volunteers except that some antitumor drugs are targeted to real patients^8^. Unlike patient-subjects participating in clinical trials, healthy volunteers for drug development trials and other research are exposed to risk and discomfort without any expectation of health benefits, and healthy volunteers may be expected to participate in clinical trials for financial reward^9^. To our surprise, although hundreds of thousands of healthy volunteers are participating in a large number of clinical trials in China, there is no report about the characteristics, recognition, or attitude of Chinese healthy volunteers and their concerns of clinical trials. In this work, we designed a questionnaire and conducted a descriptive study about the characteristics of healthy volunteers along with their experience, attitude and concerns of clinical trials in Wuhan, China, and we believed that this study was particularly important for investigators to better understand Chinese healthy volunteers as a group, to protect them and promote drug development in this country.

## Results

### Sociodemographic characteristics of Chinese healthy volunteers in Wuhan

In total, 324 healthy volunteers participated in this study and completed the questionnaire from September 2021 to January 2023, and their sociodemographic characteristics were shown in Table 1.

**Table 1:** Sociodemographic characteristics of Chinese healthy volunteers.

Variable	Categories	Total (*n* = 324)	Male (*n* = 269)	Female (*n* = 55)	Statistics	p-value
Age		27.5 (24, 34)	27 (23, 33)	31 (25, 36)	*Z* = − 3.014	0.003*
Age group	18–29 years	190 (58.6)	166 (61.7)	24 (43.6)	χ^2^ = 6.291	0.043*
	30–39 years	94 (29.0)	73 (27.1)	21 (38.2)		
	40–50 years	40 (12.3)	30 (11.2)	10 (18.2)		
Education attainment	Junior high school or below	58 (17.9)	45 (16.7)	13 (23.6)	χ^2^ = 4.638	0.326
	Technical secondary school	54 (16.7)	47 (17.5)	7 (12.7)		
	Senior high school	71 (21.9)	61 (22.7)	10 (18.2)		
	Junior college	81 (25.0)	70 (26.0)	11 (20.0)		
	Undergraduate	60 (18.5)	46 (17.1)	14 (25.5)		
	Graduate	0	0	0		
Employment status	Student	24 (7.4)	22 (8.2)	2 (3.6)	χ^2^ = 3.043	0.393
	Stable job/full-time job	109 (33.6)	94 (34.9)	15 (27.3)		
	Temporary job/part-time job	109 (33.6)	87 (32.3)	22 (40.0)		
	Unemployed	82 (25.3)	66 (24.5)	16 (29.1)		
Marriage	Unmarried	230 (71.0)	206 (76.6)	24 (43.6)	χ^2^ = 23.06	< 0.001^#^
	Married	78 (24.1)	51 (19.0)	27 (49.1)		
	Divorced	16 (4.9)	12 (4.5)	4 (7.3)		
Children	0	231 (71.3)	206 (76.6)	25 (45.5)	χ^2^ = 20.943	< 0.001^#^
	1	55 (17.0)	37 (13.8)	18 (32.7)		
	2	34 (10.5)	23 (8.6)	11 (20.0)		
	3	4 (1.2)	3 (1.1)	1 (1.8)		
The old who needs support	0	253 (78.1)	211 (78.4)	42 (76.4)	χ^2^ = 7.837	0.078
	1	20 (6.2)	15 (5.6)	5 (9.1)		
	2	45 (13.9)	40 (14.9)	5 (9.1)		
	3	1 (0.3)	0 (0)	1 (1.8)		
	4	5 (1.5)	3 (1.1)	2 (3.6)		
Residential location	Wuhan	225 (69.4)	187 (69.5)	38 (69.1)	χ^2^ = 0.709	0.702
	Hubei province except Wuhan	38 (11.7)	33 (12.3)	5 (9.1)		
	other province	61 (18.8)	49 (18.2)	12 (21.8)		
Average monthly income	< 3000 RMB	55 (17.0)	42 (15.6)	13 (23.6)	χ^2^ = 13.016	0.016*
	3000–5000 RMB	115 (35.5)	87 (32.3)	28 (50.9)		
	5000–8000 RMB	118 (36.4)	107 (39.8)	11 (20.0)		
	8000–10,000 RMB	24 (7.4)	22 (8.2)	2 (3.6)		
	10,000–15,000 RMB	6 (1.9)	5 (1.9)	1 (1.8)		
	> 15,000 RMB	6 (1.9)	6 (2.2)	0 (0)		
Satisfaction with monthly income	Satisfied	32 (9.9)	27 (10.0)	5 (9.1)	χ^2^ = 0.337	0.845
	Reasonable	177 (54.6)	145 (53.9)	32 (58.2)		
	Dissatisfied	115 (35.5)	97 (36.1)	18 (32.7)		

*p < 0.05; ^#^p < 0.01.

Among the 324 volunteers, 83.2% were male, 71.0% were unmarried, 71.3% have no children, and 78.1% do not have to support the old. The median age of volunteers was 27.5 years (range 18–50; IQR: 24, 34), and 58.6% were under the age of 30 years. 69.4% of volunteers live in Wuhan, the city in which Tongji Hospital located. In terms of the education attainment, only 60 volunteers (18.5%) have the college degree, and no one has a graduate degree. In terms of the employment status, 82 (25.3%) volunteers were unemployed, 109 (33.6%) worked part-time, and 109 (33.6%) held full-time positions. Besides, nearly half (52.5%) of the volunteers have an average monthly income of less than 5000 RMB. Only 9.9% were satisfied with their income.

In order to explore whether differences of sociodemographic characteristics exist between male and female volunteers, we separated the 324 healthy volunteers into two groups by gender. As shown in Table 1, no difference was found in terms of the education attainment, employment status, residential location and satisfaction with monthly income between the two groups. However, the median age of female volunteers was significantly older than that of the male volunteers (31 vs. 27, p = 0.003). Besides, significantly higher proportions of volunteers being married (p < 0.001), having more children (p < 0.001) and lower income (p = 0.001) were observed in female volunteers than those of the male.

### Chinese healthy volunteers’ experience of participating in clinical trials

As shown in Table 2, 267 healthy volunteers (82.4%) have participated in 1 ~ 3 clinical trials. The majority of volunteers (91%) thought that participating in trials would not greatly influence their current work. Most volunteers (65.1%) chose not to let the family know about their participation in clinical trials, and 80 volunteers’ family (24.7%) opposed their participation, double the number of volunteers (10.2%) who received support from their family. Besides, the majority of volunteers (95.4%) in this study thought that they could well or roughly understand the protocol research content before participating in one trial.

**Table 2:** Experience of Chinese healthy volunteers participating in clinical trial.

Question	Categories	Total (*n* = 324)	Male (*n* = 269)	Female (*n* = 55)	Statistics	p-value
Numbers of clinical trials participated already	1 ~ 3	267 (82.4)	222 (82.5)	45 (81.8)	χ^2^ = 0.938	0.91
	4 ~ 6	38 (11.7)	30 (11.2)	8 (14.5)		
	7 ~ 10	14 (4.3)	12 (4.5)	2 (3.6)		
	10 ~ 15	3 (0.9)	3 (1.1)	0 (0)		
	> 15	2 (0.6)	2 (0.7)	0 (0)		
The impact of participating in clinical trials on work	No influence	178 (54.9)	145 (53.9)	33 (60.0)	χ^2^ = 1.933	0.342
	Slight influence	143 (44.1)	122 (45.4)	21 (38.2)		
	Great influence	3 (0.9)	2 (0.7)	1 (1.8)		
Family support	Family did not know	211 (65.1)	177 (65.8)	34 (61.8)	χ^2^ = 0.324	0.850
	Support	33 (10.2)	27 (10.0)	6 (10.9)		
	Did not support/oppose	80 (24.7)	66 (24.2)	15 (27.3)		
Degree of understanding of the protocol research content before participating in the trial	Good understanding	129 (39.8)	108 (40.1)	21 (38.2)	χ^2^ = 0.35	0.96
	Rough understanding	180 (55.6)	148 (55.0)	32 (58.2)		
	Do not understand very well	12 (3.7)	10 (3.7)	2 (3.6)		
	Do not understand at all	3 (0.9)	3 (1.1)	0 (0)		
Age when participated in the first trial		27 (23, 33)	26 (22, 32)	30 (25, 36)	*Z* = − 3.346	0.001
Employment status when participated in the first trial	Students	32 (9.9)	27 (10.0)	5 (9.1)	χ^2^ = 0.164	0.983
	Stable job/full-time job	103 (31.8)	85 (31.6)	18 (32.7)		
	Temporary job/part-time job	117 (36.1)	98 (36.4)	19 (34.5)		
	Unemployed	72 (22.2)	59 (21.9)	13 (23.6)		
Information source of the first trial	Friends	158 (48.8)	124 (46.1)	34 (61.8)	χ^2^ = 4.619	0.197
	Subject recruitment company	105 (32.4)	91 (33.8)	14 (25.5)		
	Recruitment advertisement of hospital	35 (10.8)	30 (11.2)	5 (9.1)		
	Online media	26 (8.0)	24 (8.9)	2 (3.6)		
Satisfaction with payment in the first trial	Satisfied	94 (29.0)	81 (30.1)	13 (23.6)	χ^2^ = 1.395	0.498
	Reasonable	189 (58.3)	153 (56.9)	36 (65.5)		
	Dissatisfied	41 (12.7)	35 (13.0)	6 (10.9)		
Payment from participating in trials of the last year	< 5000 RMB	104 (32.1)	90 (33.5)	14 (25.5)	χ^2^ = 5.814	0.295
	5000–10,000 RMB	127 (39.2)	98 (36.4)	29 (52.7)		
	10,000–15,000 RMB	54 (16.7)	48 (17.8)	6 (10.9)		
	15,000–20,000 RMB	23 (7.1)	20 (7.4)	3 (5.5)		
	20,000–30,000 RMB	12 (3.7)	10 (3.7)	2 (3.6)		
	> 30,000 RMB	4 (1.2)	3 (1.1)	1 (1.8)		
Ratio of trial revenue to total income in the last year	< 20%	226 (69.8)	188 (69.9)	38 (69.1)	χ^2^ = 0.393	0.932
	20–50%	80 (24.7)	66 (24.5)	14 (25.5)		
	50–80%	16 (4.9)	13 (4.8)	3 (5.5)		
	80–100%	2 (0.6)	2 (0.7)	0 (0)		
Will you continue to participate in trials in the next two years	Yes	64 (19.8)	54 (20.1)	10 (18.2)	χ^2^ = 2.968	0.237
	Maybe	237 (73.1)	193 (71.7)	44 (80.0)		
	No	23 (7.1)	22 (8.2)	1 (1.8)		
When to stop participating in trials	Not considered at present	174 (53.7)	145 (53.9)	29 (52.7)	χ^2^ = 10.39	0.121
	When have steady income	79 (24.4)	63 (23.4)	16 (29.1)		
	When have a couple	10 (3.1)	10 (3.7)	0 (0)		
	When get married	21 (6.5)	19 (7.1)	2 (3.6)		
	When have child	2 (0.6)	0 (0)	2 (3.6)		
	When get old or sick	20 (6.2)	16 (5.9)	4 (7.3)		
	After the present trial	5 (1.5)	4 (1.5)	1 (1.8)		
	When get busy	13 (4.0)	12 (4.5)	1 (1.8)		

*p < 0.05; ^#^p < 0.01.

Because the first trial that volunteer participated in is impressive and important to one’s willingness to participate in the future, several questions about the experience of volunteers’ first trial were designed in the survey. When the volunteers participated in their first trials, the median age was 27 (range 18–50; IQR: 23, 33); 72 (22.2%) volunteers were unemployed, 117 (36.1%) worked part-time, and 103 (31.8%) held full-time positions. Besides, 48.8% volunteers got the information of their first trial from friends, 32.4% from volunteer recruitment company, and 10.8% from phase I clinical trial unit. Regarding the satisfaction with financial reward from their first trial, 87.3% answered ‘reasonable’ (58.3%) or ‘satisfied’ (29.0%), only 12.7% of volunteers said they were dissatisfied.

In terms of the trial payment in the last year, the majority of volunteers (39.2%) got 5000–10,000 RMB. The ratio of trial payment to total income in the last year was below 20% in most volunteers (69.8%), and was ranging from 20 to 50% in about a quarter of total volunteers (24.7%). 19.8% volunteers said they would continue to participate in trials within two years, 73.1% would participate if appropriate, and only 7.1% volunteers would not participate in trials within two years. Additionally, more than half of volunteers (53.7%) had not consider when to stop participating in trials, and 24.4% volunteers said they would stop participating when they have a steady income.

### Chinese healthy volunteers’ attitude and concern of participating in clinical trials

We believe that getting to know the healthy volunteers’ thought about clinical trials is important to their compliance and implementation of clinical trials, thus five ranking questions were designed to identify their attitude and concern of participating in clinical trials (Table 3). In these ranking questions, 1 means most important, 2 means the second most important; as the ranking number increases, the importance decreases.

**Table 3:** Attitude and concern of Chinese healthy volunteers participating in clinical trials.

Question	Categories	Total (*n* = 324)	Male (*n* = 269)	Female (*n* = 55)
Ranking of the main source of clinical trial information	Recruitment advertisement of hospital	2 (1, 3)	2 (1, 3)	3 (2, 3)
	communication with friend or other subjects	2 (2, 3)	3 (2, 3)	2 (2, 3)
	information from subject recruitment company	1 (1, 2)	1 (1, 2)	1 (1, 1)
Ranking of your motivation for participating in clinical trials	To get payment	1 (1, 2)	1 (1, 2)	1 (1, 2)
	to access free health checkups	2 (2, 3)	2 (2, 4)	2 (2, 3)
	to help new drug development	3 (3, 5)	4 (3, 5)	3 (3, 4)
	to accompany friends or classmates	6 (4, 8)	6 (4, 8)	6 (4, 9)
	to help other people, especially patients	4 (3, 6)	4 (3, 6)	4 (3, 5)
	curiosity	6 (4, 7.75)	6 (4, 7)	6 (5, 8)
	to meet new people	7 (6, 8)	7 (6, 8)	7 (6, 8)
	to go to different places	7 (6, 8)	7 (6, 8)	7 (6, 8)
	to learn new things and knowledge	7 (5, 9)	6 (5, 9)	7 (5, 8)
Ranking of your main consideration whether to participate in one particular clinical trial or not	The amount of payment	2 (1, 3)	2 (1, 3.5)	2 (1, 3)
	the location of phase I clinical trial unit	4 (2, 5)	3 (2, 5)	4 (3, 6)
	the convenience of transportation	4 (3, 6)	4 (3, 5)	4 (3, 7)
	arrangement of personal time	3.5 (2, 5)	3 (2, 5)	4 (2, 6)
	management of the phase I clinical trial unit	6 (5, 8)	6 (5, 8)	6 (5, 7)
	potential risk of test drug	3 (1, 6)	4 (2, 6)	2 (1, 6)
	free access to certain health checkups	7 (5, 7.75)	7 (6, 7)	7 (5, 8)
	fasting or fed trials	8 (7, 8)	8 (7, 8)	8 (5, 8)
	strictness of the inclusion and exclusion standard	8 (6, 9)	8 (5.5, 9)	8 (6, 9)
Ranking of your main consideration about whether the amount of trial payment meets your expectation	The total days of one clinical trial	2 (1, 3)	1 (1, 3)	2 (1, 4)
	the days of staying at the phase I clinical trial unit	3 (2, 5)	3 (2, 4)	4 (2, 6)
	the times of follow-up visit to phase I clinical trial unit	4 (3, 6)	4 (3, 5)	5 (3, 7)
	the times of blood collection	4 (3, 5)	4 (3, 5)	4 (2, 5)
	the volume of blood collection	5 (4, 6.75)	5 (4, 7)	5 (3, 6)
	transportation fee	7 (5, 8)	7 (5, 8)	7 (5, 8)
	the payment of similar projects at other phase I clinical trial units	7 (5, 8)	7 (5, 8)	7 (5, 8)
	the kind of test drug	6 (2, 8)	6 (3, 8)	3 (1, 7)
	the income level of the city where located the phase I clinical trial unit	9 (7, 9)	9 (7, 9)	9 (7, 9)
Ranking of your primary concern at phase I clinical trial unit	The food, e.g. variety, taste, and portion size	3 (2, 5)	3 (2, 5)	3 (2, 5)
	hardware conditions, such as the number of accommodation, and the toiletry condition	4 (2, 5)	4 (2, 5)	4 (2, 5)
	soft conditions, such as the cleanliness, environment, and recreational facilities	4 (3, 6)	4 (3, 6)	5 (3, 6)
	the signal of wifi or communication	5 (4, 7)	5 (4, 7)	6 (4, 7)
	the strict degree of management, such as whether turning off the light for rest or allowing express delivery	6 (4, 8)	6 (4, 8)	7 (4, 8)
	the professional standard of the researcher	3 (1, 6)	3 (1, 6)	2 (1, 6)
	the researchers’ attitude toward the volunteers	3 (2, 6)	3 (2, 6)	2 (2, 6)
	the living habits and conduct of the roommates	7 (4, 8)	7 (4, 8)	7 (5, 8)

#### Information sources of clinical trials

Among three information sources of clinical trials, volunteer recruitment company was considered to be the most important one in Chinese healthy volunteers, and the result was different from the main information source of their first clinical trial (friends). Besides, the recruitment advertisement from the hospital is the second important information source of clinical trial in total and male volunteers. However, female volunteers thought that communication with friends or other subjects was more important than recruitment advertisement of hospital.

#### Motivations for participating in clinical trials

Among nine motivations to participate in clinical trials, getting payment was the most important one in Chinese healthy volunteers, the same as their counterparts in many other countries^8,10,11^. Besides, accessing free health checkups and helping the development of new drugs were also important motivations in total, male and female volunteers.

#### Factors which influence whether or not to participate in a clinical trial

Among nine factors that influence whether or not to participate in a clinical trial, the amount of trial payment is the most important one in Chinese healthy volunteers. Besides, the potential risk of test drug and the arrangement of personal time are also important in total and female volunteers. However, male volunteers thought that the location of phase I clinical trial unit has a higher priority compared with the potential risk of test drug.

#### Factors which influence whether the amount of trial payment meets one’s expectation

Among nine factors which influence whether the amount of trial payment meets one’s expectation, the total days of clinical trial is the most important evaluation criterion in Chinese healthy volunteers. Besides, the days of staying at the phase I clinical trial unit and the times of blood collection are also important considerations in total and male volunteers. However, female volunteers considered that the kind of test drug has a higher priority than the days of staying at the phase I clinical trial unit and the times of blood collection.

#### Concerns at phase I clinical trial unit

Among eight concerns at a phase I clinical trial unit, the professional standard of researchers is the most important one among Chinese healthy volunteers. Besides, the food and the researchers’ attitude are also very important in total, male and female volunteers. However, for female volunteers, the researchers’ attitude toward them is more important than the food at a clinical trial unit.

### Sociodemographic characteristics associated with poorly understanding of the protocol research content before participating in the trial

Some scholars believed that in resource-limited settings, healthy volunteers are most often poor people with low literacy levels who might not understand the risks they may be taking and are in no position to refuse financial incentives^12^. As mentioned in Table 2, 129 and 180 volunteers in this study believed that they had good or rough understanding of the protocol research content before participating in the trial, while 12 and 3 ones thought that they did not understand very well or did not understand at all before participating in one trial. To explore whether certain sociodemographic characteristics were associated with poorly understanding before participating in one trial, the binary logistic regression was applied. As shown in Table 4, given the high level of understanding of the protocol research content, no sociodemographic characteristic was found to be associated with poorly understanding of the protocol research content before participating in the trial with a p value less than 0.05 in this study.

**Table 4:** Associations between sociodemographic variables and the risk of poorly understanding of protocol research content before participating in the trial.

Variables	Univariate analysis		Multivariate analysis	
	OR (95% CI)	p-value	OR (95% CI)	p-value
Gender				
Male	1.346 (0.295, 6.139)	0.701		
Female	1			
Age	0.994 (0.928, 1.065)	0.865		
Education attainment				
Junior high school or below	1			
Technical secondary school	0.333 (0.034, 3.301)	0.348		
Senior high school	0.731 (0.117, 4.548)	0.737		
Junior college	0.838 (0.163, 4.315)	0.833		
Undergraduate	1.520 (0.364, 6.339)	0.566		
Employment status				
Student	0.745 (0.147, 3.791)	0.723		
Stable job/full-time job	2.086 (0.536, 8.120)	0.289		
Temporary job/part-time job	1.145 (0.114, 11.538)	0.909		
Unemployed	1			
Number of children	0.695 (0.289, 1.676)	0.418		
The old who needs support	0.332 (0.069, 1.591)	0.168		
Average monthly income				
< 3000 RMB	1			
3000–5000 RMB	0.288 (0.025, 3.317)	0.318		
5000–8000 RMB	0.324 (0.033, 3.165)	0.333		
8000–10,000 RMB	0.130 (0.011, 1.487)	0.101		
10,000–15,000 RMB	0.217 (0.012, 4.094)	0.308		
> 15,000 RMB	0.00	0.999		

*NS* no statistical significance.

*p < 0.05, ^#^p ≤ 0.1.

## Discussion

In this study, the sociodemographic characteristics of Chinese healthy volunteers along with their experience, attitude and concern of clinical trials were reported for the first time. Four important findings emerged from our data: (1) most volunteers are young, single, and less educated men in China; (2) some differences existed between the Chinese male and female healthy volunteers, including the sociodemographic characteristics along with their attitude and concern of clinical trials; (3) no sociodemographic characteristic was associated with poorly understanding of the protocol research content; (4) more support from society/family and more positive media reports about the participation of healthy volunteers in clinical trials are badly needed in China.

First, young, single and less educated men constituted the majority of healthy volunteers in China. In this study, among the 324 healthy volunteers, 249 ones were male (83.2%). The median age of male volunteers was 27, which was significantly less than that of female volunteers. Besides, the age distribution of the male Chinese healthy volunteers was very similar to that of their counterparts in South Korea^8^. Additionally, only 19.0% of the male volunteers were married, and the proportion was significantly less than that of female volunteers. In terms of the education, only 17.1% of the Chinese male healthy volunteers obtained the undergraduate degree, and this proportion was much less than their counterparts in South Korea (89.2%)^8^, Belgium (58.0%)^11^, Singapore (69.3%)^11^, and US (67.1%)^11^.

Second, some differences between male and female healthy volunteers were observed for the first time. In this study, the median age of female volunteers was 31, which was significantly older than that of the male volunteers (31 vs. 27, p = 0.003). Additionally, significantly higher proportion of volunteers being married (p < 0.001), having more children (p < 0.001) and lower income (p = 0.001) were observed in female volunteers than those of the male. Therefore, female healthy volunteers are supposed to face more challenges and afford more pressure in life than the male ones in China. Besides, although the attitude and concern of clinical trials were almost identical among the male and female healthy volunteers, several differences existed as below: (a) when asked about the information sources of clinical trials, female volunteers thought that communication with friends or other subjects was more important than recruitment advertisement of hospital; (b) when asked about the factors which influence whether or not to participate in a clinical trial, female volunteers thought that the potential risk of test drug has a higher priority compared with the location of phase I clinical trial unit; (c) when asked about the factors which influence whether the amount of trial payment meets one’s expectation, female volunteers considered that the kind of test drug has a higher priority than the days of staying at the phase I clinical trial unit and the times of blood collection; (d) when asked about the concerns at phase I clinical trial unit, female volunteers considered that the researchers’ attitude toward them is more important than the food at a clinical trial unit. From these findings, female healthy volunteers are supposed to be more cautious about the clinical trials and more concerned about their health and feelings than the male volunteers in China^13^.

Third, no sociodemographic characteristic was found to be associated with poorly understanding of the protocol research content. Healthy volunteers in resource-limited settings are thought to be poor people with low literacy levels who might not understand the risks they may be taking and are in no position to refuse financial incentives^12^. However, in this study, no sociodemographic characteristic was found to be associated with poorly understanding of the protocol research content, which could be explained by the following reasons: (a) in this study, understanding of the protocol research content was the volunteers’ subjective perception, but not objectively evaluted, which might lead to the relatively high level of understanding (95.4%); given the high level of understanding of the protocol research content, it was not easy to find sociodemographic characteristic associated with poorly understanding of the protocol research content. Further study is needed to explore the association between sociodemographic characteristic of healthy volunteers and objectively evaluated understanding of the protocol research content; (b) good and careful informed consent process offered by the researchers in Tongji Hospital maybe improved the volunteers’ understanding of the protocol research content.

Fourth, more support from society/family and more positive media reports about the participation of healthy volunteers in clinical trials are badly needed in China. As it is known, the continued participation of volunteers in clinical studies, particularly phase I trials, is critical to advances in healthcare^14^. Regrettably, in China, there are few positive reports about the participation of healthy volunteers in clinical trials in the media. However, it is easy to find out negative media reports^15^ about horrible phase I failures (such as the ‘disasters’ in France^16^ and UK^17^) or healthy volunteers sacrificing their health for money^8,18^. Like their Korean counterparts named ‘Maruta’, which means a human subject victimized by one of the Japanese bacteriological forces during the Second World War^8^, healthy volunteers are often called little white mice in the media of China, which also have a negative meaning. We think that is why 65.1% of 324 volunteers chose not to let the family know about their participation in clinical trials, and 24.7% of the volunteers’ family opposed their participation, double the number of volunteers (10.2%) who received support from their family. The result of a Korean study showed that 76.9% of people who opposed the trial did not have previous experience in clinical trials, which indicated that there may be negative views due to lack of information^8^. Therefore, increasing the popularization of science and positive publicity of clinical trials to the public may be useful measures to gain more support from society/family for healthy volunteers in China.

Although this study was limited to phase I/BE study participants and only healthy volunteers who have participated in clinical trials in Wuhan, it has some highlights compared with the results of similar studies in other nations. Firstly, the sample size in this study (*n* = 324) was greater than most previous surveys in other nations, including South Korea^8^ (*n* = 121), Belgium^19^ (*n* = 203), Singapore^19^ (*n* = 139) and US^19^ (*n* = 202). Secondly, five ranking questions were designed to identify the healthy volunteers’ attitude and concern of participating in clinical trials, and we believed that the median ranking may provide the most comprehensive picture of participants’ attitudes toward participating in clinical trials. Thirdly, to our best knowledge, the differences found in this work between the male and female healthy volunteers were reported for the first time.

## Conclusions

This study revealed four important findings: (1) The majority of healthy volunteers in Wuhan, China were young, single and less educated men; (2) Female volunteers are supposed to face more challenges and pressure than their male counterparts in this country; (3) No sociodemographic characteristic was found to be associated with poorly understanding of the protocol research content, which was not objectively evaluated; (4) More support from society/family and more positive media reports about clinical trials are badly needed. These findings would help us to get a better understanding of healthy volunteers as a group for protecting them and promoting drug development in China.

## Methods

### Study design

This study is a descriptive study about the characteristics of healthy volunteers along with their experience, attitude and concern of clinical trials in China, which was approved by the Ethics Committee of Tongji Hospital (No. TJ-IRB20210379) and conducted according to the Helsinki Declaration and the ICH GCP. This cross-sectional survey was performed using convenience sampling between September 2021 and January 2023 for more than 300 volunteers. Healthy volunteers participating in clinical trials at the phase I clinical trial unit of Tongji Hospital, one of the top ten hospitals in China, were invited to participate in this survey.

### Questionnaire

The questionnaire survey form was shown in the Appendix. Generally, this survey questions covered three domains: (a) sociodemographic characteristics, including age, gender, income, education, employment, location of residence, and previous research experience; (b) experience of participating in clinical trials; (c) attitude and concerns of participating in clinical trials. We pilot tested the questionnaire with 20 individuals and revised question stems and response formats accordingly prior to its administration. Then the questionnaire was reviewed by two subject expert in Tongji Hospital for validity, and retest method was used to determine reliability in 10 of the above 20 individuals.

### Ethics approval and consent

This study was approved by the Ethics Committee of Tongji Hospital (No. TJ-IRB20210379) and conducted according to the Helsinki Declaration and the ICH GCP. Healthy volunteers participating in clinical trials at the phase I clinical trial unit of Tongji Hospital were invited to participate in this survey. Sufficient information and explanatory notes were provided to allow volunteers to determine whether they would participate in the study or not. The survey was carried out anonymously after volunteers provided signed informed consent, and the researchers would provide help and explanation if volunteers have problems or doubts about the content of the questionnaire survey form. Data was treated confidentially. All personal identifiers have been removed in the paper to prevent identification of personnel and their workplaces.

### Data analysis

Analyses were performed using IBM SPSS Statistics 23 (Armonk, NY IBM Corp). The distribution of continuous data was tested with the Kolmogorov–Smirnov test. Normally distributed variables were expressed as mean ± standard deviation (SD) and compared by paired-t test. Non-normally distributed ones were expressed as median (interquartile range, IQR) and compared through the Mann–Whitney U test. Categorical variables were reported as numbers and percentages (%), and the chi-square test or Fisher’s exact test was used to detect differences between two groups. The unadjusted (univariable) and adjusted (multivariable) odd ratios (OR) both for some sociodemographic characteristics and poorly understanding of one trial were calculated using logistic regression models and presented as OR with their 95% confidence intervals (CI). All independent variables with p value < 0.1 for the association with the response variable at univariable analysis were then tested in the multivariable model. For all tests, a p value < 0.05 was considered statistically significant (Supplementary Information S1 and S2).

### Supplementary Information

Supplementary Information 1.

Supplementary Information 2.

## Data Availability

The data collected in the present study are not publicly available as they contain sensitive information, but are available from the corresponding author on reasonable request.
