# Supplementary material for: Sociodemographic characteristics of healthy volunteers along with their experience, attitude and concerns of clinical trials in Wuhan, China
Source: Sci Rep. 2023 Nov 9;13:19550. doi: 10.1038/s41598-023-46979-z (PMC10636110; doi:10.1038/s41598-023-46979-z)
Supplement: Supplementary file 1 — Supplementary Information 1. [file 41598_2023_46979_MOESM1_ESM.doc]

**问卷调查表**

**说明：本调查仅用于健康受试者人群特征及对临床试验的认知、态度和关注分析，我们将严格保护您的隐私，请您如实填写。**

| 入组号： | 填表日期： |
| --- | --- |
| 性别：¨男；¨女 | 出生日期： |
| 受教育情况：¨小学；¨初中；¨中专；¨高中；¨高职高专；¨大学本科；¨硕士研究生及以上 | |
| 目前工作情况：¨有（¨稳定工作：__________）/¨临时工作：__________）/ ¨无（¨学生/¨待业） | |
| 婚姻状况：¨未婚；¨已婚；¨离异 | |
| 子女情况：¨无；¨有（ 男； 女） | 赡养老人情况：¨无；¨有（ 位） |
| 现居住地：¨武汉；¨湖北省（除武汉）；¨外省 | |
| 近一年以来的平均月收入：🞎<3000元; ¨3000-5000元; ¨5000-8000元; ¨8000-10000元; ¨10000-15000元; 🞎> 15000元 | |
| 您对目前工作的收入是否满意：¨非常满意；¨基本满意；¨不满意 | |
|  | |
| 至今已参加药物临床试验的总数量：¨1-3项；¨4-6项；¨7-10项；¨10-15项；🞎＞15项（ ） | |
| 参加药物试验是否会影响您目前的工作：¨无影响；¨影响较小；¨影响较大 | |
| 家人是否支持您参加药物临床试验：¨家人不知道；¨支持；¨不反对/反对 | |
| 在参加临床试验前，您对研究内容和知情同意的了解程度：¨很了解；¨大致了解；¨不太了解；¨完全不了解 | |
| 第一次参加药物临床试验的年龄： | |
| 第一次参加药物临床试验当时您的工作状况：  ¨有（¨稳定工作：__________）/¨临时工作：__________）；¨无（¨学生/¨待业） | |
| 第一次参加药物临床试验的信息来源：¨朋友介绍；¨招募公司；¨临床机构广告 | |
| 第一次参加药物临床试验营养费的收入是否满意：¨满意/¨不满意 | |
| 最近1年内药物临床试验的总收入：🞎＜5000元；¨5000-10000元；¨10000-15000元；¨15000-20000元；¨20000-30000元；🞎＞30000元 | |
| 最近1年内药物临床试验收入约占总收入的比例：🞎＜20%；¨20%~50%；¨50%~80%；¨80%~100% | |
| 未来2年内，如有机会，是否会继续参加新的临床试验：¨参加；¨可能参加；¨不再参加 | |
| 是否考虑过不再参加药物临床试验：¨暂未考虑；  ¨考虑过（¨收入稳定后；¨有对象后；¨结婚后；¨生子后；¨年纪偏大后；¨其他：_______） | |
|  | |
| 目前您获悉药物试验信息的主要来源途径排序（根据重要程度在括号中填写1-3；1位最重要；随着数字的增大，重要性降低）  （ ）临床试验机构发布广告  （ ）受试者间的交流  （ ）招募公司发布信息 | |
|  | |
| 您参加药物临床试验的动机排序（根据重要程度在括号中填写1-9；1位最重要；随着数字的增大，重要性降低）  （ ）能获得营养费收入  （ ）能获得免费体检的机会  （ ）陪好朋友或同学一起参加  （ ）有助于新药的研发  （ ）药物临床试验能够帮助到他人，尤其是患者  （ ）因为好奇心  （ ）可以认识新朋友  （ ）可以到不同的地方  （ ）可以学习到新的事物和知识 | |
| 您决定参加某项临床试验的主要考虑因素排序（根据重要程度在括号中填写1-9；1位最重要；随着数字的增大，重要性降低）  （ ）营养费金额的多少  （ ）试验中心所在区域省份  （ ）来去交通的便利程度  （ ）个人的时间安排  （ ）研究中心管理严格程度  （ ）药物的潜在风险  （ ）入排标准是否严格：如烟检、B超、胸片等  （ ）能够免费获得的检查项目  （ ）空腹试验或餐后试验 | |
| 您判断营养费金额是否符合预期的主要考虑因素排序（根据重要程度在括号中填写1-9；1位最重要；随着数字的增大，重要性降低）  （ ）试验总天数  （ ）待在病房的天数  （ ）来试验中心的次数  （ ）采血次数  （ ）采血体积  （ ）交通费用  （ ）与其他基地类似项目的比较  （ ）药物类型：如抗肿瘤药物金额高于其他药  （ ）中心所在城市评价收入水平高低 | |
| 您在临床试验中心最关心的事项排序（根据重要程度在括号中填写1-8；1位最重要；随着数字的增大，重要性降低）  （ ）餐饮伙食可口程度  （ ）硬件条件（如住宿人数、洗漱）  （ ）软件条件（卫生、环境、娱乐设施）  （ ）通信、WIFI信号  （ ）管理的严格程度  （ ）试验中心研究者的专业水准  （ ）研究者对受试者的态度（ ）  （ ）同宿室友的生活习惯和品行 | |
