# Supplementary material for: Sociodemographic characteristics of healthy volunteers along with their experience, attitude and concerns of clinical trials in Wuhan, China
Source: Sci Rep. 2023 Nov 9;13:19550. doi: 10.1038/s41598-023-46979-z (PMC10636110; doi:10.1038/s41598-023-46979-z)
Supplement: Supplementary file 2 — Supplementary Information 2. [file 41598_2023_46979_MOESM2_ESM.doc]

**Questionnaire Survey Form**

**Note: This questionnaire is only used for analyzing the characteristics, recognition, attitude of Chinese healthy volunteers and their concerns of clinical trials. Your privacy will be strictly protected. Please fill it out truthfully.**

| Random number: | Date of filling: |
| --- | --- |
| Gender: 🞎 male; 🞎 female | Date of birth: |
| Education: ¨junior high school or below; ¨technical secondary school; ¨senior high school; ¨junior college; ¨vocational college; ¨undergraduate; ¨master; ¨doctor | |
| Employment status: ¨student; ¨stable job/full-time job; ¨temporary job/part-time job; ¨unemployed | |
| Marital Status: 🞎 unmarried; 🞎 married; 🞎 divorced | |
| Children: ¨none; ¨have ( boy; girl) | The old need to support: ¨none; ¨have |
| Residential location: ¨Wuhan; ¨Hubei province except Wuhan; 🞎 other province | |
| Average monthly income in the last year: 🞎<3000RMB; ¨3000-5000RMB; ¨5000-8000RMB; ¨8000-10000RMB; ¨10000-15000RMB; 🞎> 15000RMB | |
| Satisfaction with your monthly income: ¨satisfied; ¨reasonable; ¨dissatisfied | |
|  | |
| Numbers of clinical trials participated already: ¨1-3; ¨4-6; ¨7-10; ¨10-15; 🞎> 15 | |
| The impact of participating in clinical trials on your current work: ¨no influence; ¨slight influence; ¨great influence | |
| Do the family support your participation in clinical trials? ¨family did not know; ¨support; ¨did not support/oppose | |
| Degree of understanding of the protocol research content before participating in the trial: ¨good understanding; 🞎 rough understanding; 🞎 do not understand very well; 🞎 do not understand at all | |
| Age when participated in the first trial: | |
| Employment status when you participated in the first trial: ¨student; ¨stable job/full-time job; ¨temporary job/part-time job; ¨unemployed | |
| Information source of your first trial: ¨friends; ¨subject recruitment company; ¨recruitment advertisement of hospital; ¨online media | |
| Satisfaction with payment in the first trial: ¨satisfied; ¨reasonable; ¨dissatisfied | |
| Payment from participating in trials of the last year: 🞎<5000RMB; ¨5000-10000RMB; ¨10000-15000RMB; ¨15000-20000RMB; ¨20000-30000RMB; 🞎> 30000RMB | |
| Ratio of trial revenue to total income in the last year: 🞎< 20%; ¨20%~50%; ¨50%~80%; ¨80%~100% | |
| Will you continue to participate in trials in the next two years: ¨yes; ¨maybe; ¨no | |
| When to stop participating in trials: ¨not considered at present;¨when have steady income; ¨when have a couple; ¨when get married; ¨when have child; ¨when get old or sick; ¨others______ | |
|  | |
| Ranking of the main source of clinical trial information (1-3 in parentheses; 1 means most important, as the ranking number increases, the importance decreases)  ( ) Recruitment advertisement of hospital  ( ) communication with friend or other subjects  ( ) Information from subject recruitment company | |
| Ranking of your motivation for participating in clinical trials (1-9 in parentheses; 1 means most important, as the ranking number increases, the importance decreases)  ( ) to get payment  ( ) to access free health checkups  ( ) to help new drug development  ( ) to accompany friends or classmates  ( ) to help other people, especially patients  ( ) curiosity  ( ) to meet new people  ( ) to go to different places  ( ) to learn new things and knowledge | |
| Ranking of your main consideration whether to participate in one particular clinical trial or not (1-9 in parentheses; 1 means most important, as the ranking number increases, the importance decreases)  ( )the amount of payment  ( )the location of phase I clinical trial unit  ( )the convenience of transportation  ( )arrangement of personal time  ( )management of the phase I clinical trial unit  ( )potential risk of test drug  ( )free access to certain health checkups  ( )fasting or fed trials  ( )strictness of the inclusion and exclusion standard | |
| Ranking of your main consideration about whether the amount of trial payment meets your expectation (1-9 in parentheses; 1 means most important, as the ranking number increases, the importance decreases)  ( ) the total days of one clinical trial  ( ) the days of staying at the phase I clinical trial unit  ( ) the times of follow-up visit to phase I clinical trial unit  ( ) the times of blood collection  ( ) the volume of blood collection  ( ) transportation fee  ( ) the payment of similar projects at other phase I clinical trial units  ( ) the kind of test drug  ( ) the income level of the city where located the phase I clinical trial unit | |
| Ranking of your primary concern at phase I clinical trial unit (1-8 in parentheses; 1 means most important, as the ranking number increases, the importance decreases)  ( ) the food, eg variety, taste, and portion size  ( ) hardware conditions, such as the number of accommodation, and the toiletry condition  ( ) soft conditions, such as the cleanliness, environment, and recreational facilities  ( ) the signal of wifi or communication  ( ) the strict degree of management, such as whether turning off the light for rest or allowing express delivery  ( ) the professional standard of the researcher  ( ) the researchers’ attitude toward the volunteers  ( ) the living habits and conduct of the roommates | |
